# Une occlusion intestinale aiguë inhabituelle

**DOI:** 10.11604/pamj.2015.20.296.6647

**Published:** 2015-03-26

**Authors:** Ammar Mahmoudi, Abdelaziz Hamdi

**Affiliations:** 1 Service de Chirurgie Générale et Digestive, CHU Fattouma Bourguiba de Monastir, Tunisie

**Keywords:** Colorectal, rectum, colon, corps étranger, occlusion intestinale aiguë, méthodes d´extraction, voie anale, laparotomie, Colorectal, rectum, colon, foreign body, acute intestinal obstruction, extraction methods, anally, laparotomy

## Image en medicine

**Figure 1:**
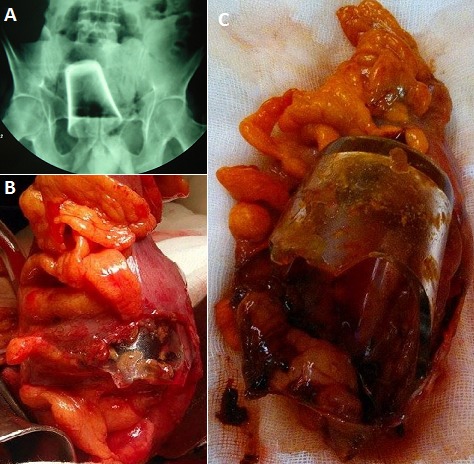
A): radiographie de l'abdomen sans préparation montrant un corps étranger (verre inversé) se projetant au niveau du pelvis responsable d'une occlusion intestinale de type colique; B): vue opératoire montrant la perforation du sigmoïde par la partie du verre cassé; C): pièce opératoire de la partie sigmoïdienne réséquée avec le corps étranger cassé

L'insertion de corps étrangers intrarectal reste dans notre contexte, une curiosité et un tabou. Elle est le plus souvent volontaire par trouble du comportement, pour des fins sexuelles (érotisme ou agressions), pour auto-traiter une constipation ou pour dissimuler l'objet (drogues, etc). Elle est rarement accidentelle. Ces corps étrangers peuvent être de nature très diverse et insolite. L'extraction de l'objet par voie anale permet quand elle est réalisable, d’éviter la chirurgie qui s'impose en cas d’échec ou de complications. Nous rapportons l'observation d'un homme âgé de 62 ans bronchitique chronique, qui avait consulté pour syndrome occlusif évoluant depuis deux jours. A l'examen, l'abdomen était distendu tympanique, le toucher rectal avait perçu un corps étranger intrarectal. A la reprise de l'interrogatoire, les circonstances étaient non précisées. La radiographie de l'abdomen sans préparation avait confirmé l'occlusion colique et le corps étranger (verre à base haute). La tentative d'extraction par voie anale sous anesthésie générale a été un échec du fait du siège haut et du caractère impacté dans la paroi entrainant une perforation rectale. Au cours de la manipulation, le verre avait cassé, et le bout restant avait échappé et migré vers le haut. Par laparotomie, il existait une perforation sigmoïdienne à berges nécrosées. Il a été réalisé une intervention de Hartmann emportant la partie du sigmoïde perforée (contenant le corps étranger), une toilette péritonéale, un drainage et une toilette rectale. Les suites opératoires ont été marquées par une surinfection broncho-pulmonaire sévère aboutissant au décès à j10 malgré une antibiothérapie et une réanimation adéquates. Figure 1

